# New Therapeutic Targets for Mood Disorders

**DOI:** 10.1100/tsw.2010.65

**Published:** 2010-04-13

**Authors:** Rodrigo Machado-Vieira, Giacomo Salvadore, Nancy DiazGranados, Lobna Ibrahim, David Latov, Cristina Wheeler-Castillo, Jacqueline Baumann, Ioline D. Henter, Carlos A. Zarate

**Affiliations:** ^1^ Experimental Therapeutics, Mood and Anxiety Disorders Research Program, National Institute of Mental Health, National Institutes of Health, Bethesda, MD, USA; ^2^ Institute and Department of Psychiatry, LIM-27, University of Sao Paulo, Brazil

**Keywords:** bipolar disorder, mania, depression, treatment, mood, trial, therapeutic, target

## Abstract

Existing pharmacological treatments for bipolar disorder (BPD) and major depressive disorder (MDD) are often insufficient for many patients. Here we describe a number of targets/compounds that clinical and preclinical studies suggest could result in putative novel treatments for mood disorders. These include: (1) glycogen synthase kinase-3 (GSK-3) and protein kinase C (PKC), (2) the purinergic system, (3) histone deacetylases (HDACs), (4) the melatonergic system, (5) the tachykinin neuropeptides system, (6) the glutamatergic system, and (7) oxidative stress and bioenergetics. The paper reviews data on new compounds that have shown antimanic or antidepressant effects in subjects with mood disorders, or similar effects in preclinical animal models. Overall, an improved understanding of the neurobiological underpinnings of mood disorders is critical in order to develop targeted treatments that are more effective, act more rapidly, and are better tolerated than currently available therapies.

## INTRODUCTION

Mood disorders, such as bipolar disorder (BPD) and major depressive disorder (MDD), are severe, chronic, and disabling psychiatric disorders commonly associated with persistent subsyndromal symptoms and frequent episode relapses[1]. For instance, less than one-third of patients with MDD achieve remission after 10–14 weeks of treatment with a standard antidepressant[2]. Similarly, in BPD, few treatments have proven to be effective for acute depressive episodes or maintenance treatment[3,4]. Except for lithium, all available Food and Drug Administration (FDA)–approved treatments for BPD are either anticonvulsant or antipsychotic drugs originally developed to treat other conditions[5]. In addition, clinical and genetic studies support the notion that mood disorders are heterogeneous conditions with a wide range of clinical features. In this context, not all patients benefit from the same treatment; for instance, lower response rates are seen in subjects with rapid cycling, as well as those presenting with a greater number of psychiatric comorbidities or suicidality.

Treatment of mood disorders has typically focused on alleviating symptoms and preventing recurrence of episodes, but other clinical aspects, such as cognitive function, residual symptoms, and suicidal ideation, are also relevant to the notion of effective treatment[6]. Novel treatments are expected to address these clinical challenges, and it is critical to the health of our patients that the next generation of treatments for mood disorders be more effective, better tolerated, and act faster than currently available treatments[7].

Here, we describe studies that evaluate potential promising targets for the development of new, improved treatments for mood disorders. Specifically, we review (1) glycogen synthase kinase-3 (GSK-3) and protein kinase C (PKC), (2) the purinergic system, (3) histone deacetylase (HDAC), (4) the melatonergic system, (5) the tachykinin neuropeptides system, (6) the glutamatergic system, and (7) oxidative stress and bioenergetics (see Table 1).

**TABLE 1**
**New Therapeutic Targets in Mood Disorders: Current Evidence for Antimanic (-Like)/ Antidepressant (-Like) Effects and Effects of Standard Antidepressants/Mood Stabilizers at these Targets**

| System | Antimanic Effects | | Antidepressant Effects | | Target for Antidepressants | Target for Mood Stabilizers |
|---|---|---|---|---|---|---|
| | Clinical | Preclinical | Clinical | Preclinical | | |
| GSK-3 | No | Yes | No | Yes | Yes | Yes |
| PKC | Yes | Yes | No | Yes | Yes | Yes |
| Purinergic | Yes | Yes | No | Yes | No | Yes |
| Histone deacetylases (HDACs) | No | No | No | Yes | No | Yes |
| Melatonergic | No | No | Yes | Yes | Yes | Yes |
| Tachykinin neuropeptide | No | No | Yes | Yes | Yes | Yes |
| Glutamatergic | No | Yes | Yes | Yes | Yes | Yes |
| Oxidative stress and bioenergetics | No | No | Yes | Yes | Yes | Yes |

GSK-3, glycogen synthase kinase 3; PKC, protein kinase C.

This paper reviews data on new compounds that have shown antimanic or antidepressant effects in subjects with mood disorders, or similar effects in preclinical animal models. It is important to mention at the outset that the extrapolation of animal studies to humans requires cautious interpretation.

## GLYCOGEN SYNTHASE KINASE-3 (GSK-3) AND PROTEIN KINASE C (PKC)

### GSK-3

GSK-3 is a multifunctional and highly active serine/threonine kinase that regulates diverse signaling pathways (e.g., the phosphoinositide 3-kinase [PI3K] pathway, the Wnt pathway, protein kinase A (PKA), and PKC). In general, increased activity of GSK-3 is proapoptotic, whereas inhibiting GSK-3 prevents apoptosis. GSK-3 (isoforms α and β) is an important regulator of glycogen synthesis, gene transcription, synaptic plasticity, apoptosis (cell death), cellular structure, and resilience[8]. It has been suggested that GSK-3 regulates behavior by affecting β-catenin, glutamate receptors, circadian rhythms, and serotonergic neurotransmission (reviewed in Beaulieu et al.[9]). All of these have been implicated in the pathophysiology of severe mood disorders.

Lithium has been shown to target GSK-3β in several paradigms[9,10,11]. Lithium also induces neurotrophic and neuroprotective effects in rodents, partly due to GSK-3β inhibition (reviewed in Gould and Manji[12]). Mice overexpressing a constitutively active form of brain GSK-3β have increased locomotor activity and decreased habituation in the open field test. In contrast, and similar to lithium's effects, pharmacologic or genetic inhibition of GSK-3β significantly decreased dopamine-dependent locomotor hyperactivity and induced similar molecular changes[9,13].

In addition, the GSK-3 inhibitor AR-A014418 was shown to have both antidepressant-like effects in the forced swim test and antimanic-like effects in the D-amphetamine hyperlocomotion model[14,15]. Valproate was initially reported to inhibit GSK-3β activity in SH-SY5Y cells[16,17], but these effects have not been confirmed in neuronal cells[18]. With respect to carbamazepine, this drug was reported to be involved in signal transduction of cyclic adenosine monophosphate (cAMP) second messenger systems, but no effect on Akt/GSK-3β has been reported to date[19]. For other agents effective in mood disorders, few studies have been carried out investigating either cell survival or potentiation of cell proliferation, but results suggest that this signaling pathway is not shared by all mood stabilizers[20,21].

Taken together, these findings suggest that this class of compounds has relevant antimanic and antidepressant effects. However, inhibition of GSK-3β is associated with some limitations due to its involvement with diverse pathways that contain multiple substrates that may lead to side effects or toxicity[22]. At present, no blood brain barrier–penetrant GSK-selective inhibitors have been clinically tested. Proof-of-principle studies with selective and safe GSK-3β inhibitors are needed in order to establish the potential safety and therapeutic relevance of this target in mood disorders.

## PKC Signaling Cascade

Diverse studies support the involvement of PKC and its substrates in the pathophysiology and therapeutics of BPD[23,24,25,26,27,28]. PKC plays an important role in regulating neuronal excitability, neurotransmitter release, and long-term alterations in gene expression and plasticity. Its isoforms differ in their structure, subcellular localization, tissue specificity, mode of activation, and substrate specificity[29].

Diverse studies also show that PKC is regulated by the mood stabilizers lithium and valproate. In addition, two recent clinical trials provide further evidence for the involvement of this system in bipolar mania[30,31]. Although well known for its antiestrogenic properties, tamoxifen is also a potent PKC inhibitor at high concentrations. For instance, one preclinical study found that it decreased amphetamine-induced hyperactivity in a large open field, a well-validated preclinical measure for studying mood-related behaviors compared with the smaller standard activity monitors[32]. In a single-blind study, tamoxifen was found to have significant antimanic effects in five of seven BPD subjects[33]. Another 4-week, three-arm, double-blind, placebo-controlled, add-on study involving 13 women compared tamoxifen ($n = 5$) to medroxyprogesterone acetate ($n = 4$) and placebo ($n = 4$). Subjects in the tamoxifen group had a significantly greater decrease in manic and positive psychotic symptoms compared to the placebo group. All patients were receiving concomitant treatment that consisted of either lithium (0.8–1.0 mmol/l) or valproate[34]. These initial results were confirmed in two recent, 3-week, double-blind, placebo-controlled, monotherapy studies[30,31]. The study by Zarate and colleagues tested higher doses of up to 140 mg/day and ratings were obtained daily during the first week, thus permitting the assessment of early antimanic effects[31]. Yildiz and colleagues used doses of up to 80 mg/day and weekly ratings were obtained, but because of the increased sample size, they were able to both separate the effects of tamoxifen on other specific domains (e.g., psychosis and depression) and to perform multivariate regression models of response[30]. In all cases, the antimanic effects of tamoxifen were not related to its sedative effects, and no increased risk of depression was observed; however, it is possible that some of tamoxifen's antimanic effects may be due to its antiestrogenic properties (see Goldstein[35]). It is also interesting to note that other drugs tested in BPD, such as omega-3 fatty acids and verapamil, inhibit PKC activity, which reinforces the role of this target in drug development for BPD. For instance, verapamil had significant antimanic effects when combined with lithium in a double-blind, randomized study[36]. There is preliminary evidence that PKC activation may be a potential target for developing novel antidepressants[37]. Large controlled studies with selective PKC inhibitors are necessary in order to confirm the potentially important role of PKC in developing novel therapeutics for mood disorders.

## THE PURINERGIC SYSTEM

Purinergic neurotransmission is mediated by adenosine triphosphate (ATP) and adenosine, and is involved in regulating cognition, sleep, motor activity, appetite, memory, and social interaction[38]. Purines also modulate the activity of diverse neurotransmitters involved in the pathophysiology of mood disorders, such as dopamine, gamma aminobutyric acid (GABA), and serotonin[39]. In preclinical paradigms, adenosine induced antidepressant-like and anticonvulsant effects via its effects at the adenosine 1 and 2A receptors[39,40]. In animal models, adenosine agonists have sedative, anticonvulsant, antiaggressive, and antipsychotic properties, while adenosine antagonists (e.g., caffeine) increase anxiety, insomnia, and irritability[41,42]. A1 receptor agonists have been shown to limit the activating effects of caffeine, while A1 receptor antagonists induce stimulating behavioral effects similar to those of caffeine[43]. In animal models of mania, the ATP P2 receptor antagonist PPADS blocked amphetamine-induced motor hyperactivity[44].

Clinically, the involvement of purinergic system dysfunction in mood disorders has been described in diverse studies[38,45,46]. Specifically, the purinergic modulator allopurinol showed antimanic efficacy as an add-on therapy in three different clinical studies, one case report and two double-blind, placebo-controlled studies[47,48,49]. Allopurinol was significantly superior to placebo in decreasing manic symptoms in both controlled studies. Notably, a significant association was found between uric acid levels and allopurinol's antimanic effects[49]. Recent genetic investigations also suggest that a single nucleotide polymorphism at the P2RX7 gene may play a role in the pathophysiology of mood disorders[50,51]. Finally, a recent study found that never-treated subjects with BPD showed increased plasma uric acid levels during a first manic episode compared to healthy controls[52]. Further controlled studies with allopurinol as monotherapy, as well as the use of more selective purinergic agents, may further clarify the role of the purinergic system in the development of new therapeutics for mood disorders.

## HISTONE DEACETYLASES (HDACs)

Epigenetics involves the study of heritable variations in gene function that cannot be explained by modifications in DNA sequence and chromatin structure[53], mostly related to decreased DNA methylation and increased acetylation of histones; histones are small proteins that form the nucleosome core by complexing with DNA. Epigenetic changes can permanently alter gene expression, which may induce subsequent changes in behavior; however, such effects may be potentially reversible over time[53].

Histone acetylation has been considered a promising therapeutic target in mood disorders because of its ability to control epigenetic effects that regulate cognitive and behavioral processes. Histone acetylation reduces histones' affinity for DNA and is a major epigenetic regulator of gene expression for several key proteins. Thus, diverse HDAC inhibitors have been developed that could serve as novel neuroprotective agents; their ability to affect neuronal function and protection occurs largely through epigenetic mechanisms[54]. In addition, it has been suggested that central nervous system (CNS)–

penetrant HDAC inhibitors may eventually have potential therapeutic relevance in mood disorders, supposedly due to their ability to reverse dysfunctional epigenetic effects associated with early life events. In neuronal tissue, HDAC inhibitors limit histone deacetylation mostly by inactivating class I or II HDACs, thus increasing histone acetylation (for a review see Grayson et al.[55]).

In animal models of stress and depression, central infusion of an HDAC inhibitor removed the group differences in histone deacetylation, DNA methylation, and hypothalamic-pituitary-adrenal (HPA) stress responses[56]. Two other preclinical studies[57,58] described antidepressant-like effects associated with sodium butyrate, a nonspecific class I and II HDAC inhibitor. Recently, the use of two HDAC inhibitors (two selective inhibitors of class I and II HDACs) administered directly into the nucleus accumbens induced potent antidepressant-like effects in several behavioral models; furthermore, these effects were seen at the gene expression level[59]. The same study found a similar decrease in HDAC II protein expression in the nucleus accumbens of individuals with MDD.

Notably, the mood stabilizer valproate is an HDAC inhibitor, suggesting that its effects at this target may play a therapeutic role in mood stabilization, although it may also be associated with side effects such as teratogenicity or polycystic ovarian syndrome[60,61]. Also, down-regulation of reelin and GAD(67) expression in cortical interneurons in individuals with BPD may be regulated by epigenetic hypermethylation[62]. The same group noted that valproate blocked methionine-induced reelin promoter hypermethylation and reelin mRNA down-regulation, thus also improving social interaction in preclinical models[62].

Overall, the growing relevance of gene-environment interactions and early life stressors in the pathophysiology of severe mood disorders raises the intriguing possibility that regulating histone acetylation and the clinical use of HDAC inhibitors might represent a new and promising therapeutic target in the treatment of mood disorders.

## THE MELATONERGIC SYSTEM

Melatonin receptors (MT1 and MT2) are highly expressed in the brain, and induce biological effects mostly through G protein–coupled receptors. Supersensitivity to melatonin suppression by light was described in individuals with mood disorders and their unaffected offspring[63]. In addition, an association was described between a polymorphism in GPR50 (a melatonin-related receptor) and increased risk for BPD[64].

No controlled studies have evaluated the use of melatonin as a potential treatment for BPD, and case reports describe conflicting findings[65,66]. A recent, open-label, 6-week study in 21 individuals with BPD assessed the effects of agomelatine (25 mg/day), a nonselective MT1 and MT2 receptor agonist. Eighty-one percent of patients achieved significant improvement at end point, and 47% showed response during the first week of treatment[67].

In contrast, strong evidence exists that melatonergic modulators may play a key role as therapeutics for MDD. In three large, controlled, multicenter, clinical trials, agomelatine was found to be safe as well as more effective than placebo[68,69,70]; agomelatine was also associated with low rates of weight gain and sexual dysfunction, and no evidence of discontinuation syndrome[71].

In preclinical models, agomelatine had significant antidepressant-like effects in the forced swim test, the chronic mild stress test, and the learned helplessness paradigm[72,73,74]. It was also capable of resynchronizing a disrupted circadian rhythm and had circadian phase-advancement properties[75,76,77]. Agomelatine is also known to increase both norepinephrine and dopamine, and to increase cell proliferation and neurogenesis in the ventral dentate gyrus[66,78]. Thus, a growing body of evidence supports a relevant role for melatonergic modulators as therapeutics for MDD. These findings are promising, and further placebo-controlled studies evaluating their potential use in bipolar depression are necessary.

## THE TACHYKININ NEUROPEPTIDES SYSTEM

Several preclinical and clinical studies have investigated the potential therapeutic role of the tachykinin neuropeptides system in mood disorders. These neuropeptides involve substance P, neurokinin A, neurokinin B, and their respective receptors (NK1, NK2, NK3). They are G protein–coupled receptors, leading to activation of the phospholipase C (PLC), inositol trisphosphate (IP3), and diacylglycerol (DAG) signaling cascades[79]. These act as neuromodulators and neurotransmitters, and interact directly with the monoaminergic system in diverse brain areas implicated in mood regulation and emotion-processing[80].

Heterogeneous results were obtained in phase II and III clinical trials testing diverse neurokinin receptor antagonists in mood disorders. Three different NK1 receptor antagonists, MK869, L759274, and CP122721, reduced depressive symptoms compared to placebo in randomized, double-blind, phase II clinical trials; both MK869 and CP122721 were associated with fewer side effects than the active comparator (paroxetine or fluoxetine)[81,82]. However, the efficacy of MK869 was not subsequently replicated in a multisite, placebo-controlled, phase III trial[83]. Similarly, the NK3 receptor antagonist SR142801 (osanetant) was not superior to placebo or paroxetine in a 6-week phase II trial[84]. The most promising agent for the treatment of mood disorders appears to be the NK2 receptor antagonist SR48968 (*saredutant*), which showed significant antidepressant effects in MDD and is currently in phase III trials. The efficacy, tolerability, and safety of this compound were evaluated in adult and elderly patients with MDD[80].

Tachykinin antagonists also induce antidepressant-like effects in preclinical models, particularly NK2 receptor antagonists[85,86,87,88]. Neurobiological findings further suggest that individuals with MDD have increased CSF and serum substance P[80]. Finally, some agents acting as neuropeptide system antagonists appear to potentiate the therapeutic effects of selective serotonin reuptake inhibitors (SSRIs). Future studies may shed light on the potential role of this system in the therapeutics of mood disorders, particularly NK1 and NK2 antagonists.

## THE GLUTAMATERGIC SYSTEM

Recent research into the etiology of BPD suggests that altered glutamatergic activity involving a potential dysregulation in neuroplasticity and cellular resilience may play a key role in this disorder. Therefore, diverse glutamatergic modulators that target both ionotropic and metabotropic glutamate receptors have been tested in ‘‘proof of concept’’ studies in mood disorders[89].

### Ionotropic Glutamate Receptors

In preclinical studies, N-methyl-D-aspartate (NMDA) receptor antagonists have been found to have antidepressant-like effects (reviewed in Zarate et al.[90,91]). For instance, the NMDA antagonists dizocilpine (MK-801) and CGP 37849 exert significant antidepressant-like effects alone or combined with standard antidepressants[74,92,93,94,95].

Ketamine is a high-affinity NMDA receptor antagonist with a specific type of channel closure (called “trapping block”). Ketamine induces a significant presynaptic release of glutamate by enhancing the firing of glutamatergic neurons[96], and it has been proposed that ketamine’s rapid antidepressant effects (described below) are mediated by α-amino-3-hydroxyl-5-methyl-4-isoxazole-propionate (AMPA) throughput[97]. In preclinical models, ketamine induced both anxiolytic and antidepressant effects[97,98,99,100,101].

Ketamine has also been tested in clinical studies. A pilot study found improvement of depressive symptoms 72 h after ketamine infusion in treatment-resistant MDD patients[102]. More recently, a double-blind, placebo-controlled, cross-over study found that a single ketamine infusion (single-dose, 0.5

mg/kg for 40 min) in patients with treatment-resistant MDD was associated with fast (within 2 h), significant, and relatively sustained antidepressant effects (lasting 1–2 weeks)[103]. More than 70% of patients met criteria for response (50% improvement) at 24 h after infusion, and 35% showed a sustained response after 1 week. Another selective NMDA antagonist, memantine, showed no antidepressant efficacy in a double-blind, placebo-controlled, 8-week trial in 32 subjects with MDD. Trials assessing the potential usefulness of more selective subtype NMDA antagonists are under way.

AMPA receptor potentiators (also known as AMPAkines) have also been tested (reviewed in Black[104], Du et al.[105], and Miu et al.[106]). In one preclinical study, the AMPAkine Ampalex induced antidepressant-like effects during the first days of treatment[107]. Such effects may involve AMPA receptor trafficking (including receptor insertion, internalization, and delivery to synaptic sites), which critically regulates synaptic strength, as well as neural and behavioral plasticity[108].

Riluzole is FDA approved for the treatment of amyotrophic lateral sclerosis (ALS) based on its well-characterized neuroprotective properties. Riluzole activates AMPA trafficking and membrane insertion of GluR1 and GluR2 subunits, thus limiting glutamate release. Riluzole also enhances glutamate reuptake and activates synthesis of neurotrophins[109,110]. Interestingly, one preclinical study found that pretreatment with 10 mg/kg riluzole (but not 3 mg/kg) moderately reduced amphetamine-induced hyperlocomotion[111], which could suggest potential antimanic efficacy.

Clinical studies assessing riluzole's efficacy in treating both MDD and BPD have noted that riluzole induced significant antidepressant effects and was well tolerated. In one open-label study, 13 patients (68%) with MDD completed the trial and all had achieved improvement at week 6[112]. Another open-label study of 14 patients with BPD found that riluzole (100–200 mg/day) used adjunctively to lithium for 6 weeks was similarly effective[113]. Another study of 10 patients with treatment-resistant depression found that riluzole (50 mg twice daily) added to the patients' existing medications produced antidepressant effects after 1 week of treatment, with a significant decrease (36%) in Hamilton Depression Rating Scale (HAM-D) scores among completers[114]. Placebo-controlled trials with riluzole are needed to confirm these promising findings. Recent data suggest that GluR6 knock-out mice have increased motor activity in response to amphetamine challenge, with elevated risk taking and aggressive behavior; these manifestations were abolished after chronic treatment with lithium[115].

## Metabotropic Glutamate Receptors (mGluRs) and Glutamate-Glutamine Cycling

The mGluR family comprises eight receptor subtypes (mGluR1 to GluR8) classified into three groups based on their sequence homology, agonist selectivity, and second messenger systems coupled receptor. In several animal models, diverse group I mGluR1 and mGluR5 antagonists induced mood-improving effects[89]. For instance, the potent and selective mGluR5 antagonist fenobam induced anxiolytic effects, although studies evaluating its use were discontinued because of its psychostimulant effects[116]. In addition, the mGluR5-positive allosteric modulator CDPPB reversed amphetamine-induced locomotor activity in rodents, with relevance for developing therapeutics for bipolar mania[117].

The group II mGluR2 and mGluR2/3 receptors reduce excessive glutamate levels into the synapse. Diverse group II mGluR modulators (e.g., LY341495) have been tested and show a dose-dependent antidepressant-like efficacy in animal models (reviewed in Zarate et al.[91]). Similarly, group III mGluR agonists showed mood-enhancing effects in the forced swim and behavioral despair tests[116,118]. In addition, in animal models of depression, mGluR7 knock-out mice displayed an antidepressant phenotype[119].

Cytidine is a pyrimidine component of RNA that controls neuronal-glial glutamate cycling, affecting cerebral phospholipid metabolism, catecholamine synthesis, and mitochondrial function. Cytidine was studied as an add-on to valproate in a double-blind, 12-week, placebo-controlled trial evaluating 35 individuals with BPD[120]. This agent induced earlier improvement of depressive symptoms in the active compound group, and this improvement directly correlated with a decrease in midfrontal glutamate/glutamine levels. The study suggests that cytidine supplementation has therapeutic effects in BPD, potentially mediated by a decrease of cerebral glutamate/glutamine levels.

## OXIDATIVE STRESS AND BIOENERGETICS

### N-Acetyl Cysteine (NAC)

Increasing evidence suggests that oxidative stress parameters play a key role in the pathophysiology of BPD[121,122]. Altered levels of glutathione, the most abundant antioxidant substrate in all tissues, have been described in BPD subjects[123,124]. Glutathione production is regulated by its precursor, cysteine. Treatment with N-acetyl cysteine (NAC), another precursor of glutathione, increases glutathione levels. A recent, randomized, double-blind, multicenter, placebo-controlled study involving 75 patients with BPD evaluated NAC (1 g twice daily) added on to treatment as usual over 24 weeks, followed by a 4-week washout phase. By study end point, NAC showed superior antidepressant effects compared to placebo, as assessed by Montgomery Asberg Depression Rating Scale (MADRS) scores and most secondary scale scores[125]. It is interesting to note that patients were not necessarily selected for having a major depressive episode. However, there was a considerable lag in the benefits obtained, and benefits were lost shortly after discontinuing the study medication. No significant differences in side effects compared to placebo were noted. The authors hypothesized that NAC's efficacy might be due to its ability to reverse increased oxidative stress during mood episodes.

### Creatine

Creatine plays a key role in brain energy homeostasis, and its dysfunction has been implicated in BPD. Brain creatine kinase has been shown to be altered in the hippocampus in animal models of mania as well as in subjects with BPD during a manic episode[126,127]. Thus, creatine supplementation may modify brain high-energy phosphate metabolism in individuals with BPD. Recently, an open-label study in 10 treatment-resistant patients with depression (two of whom had BPD) found that 3–5 mg/day of creatine monohydrate added to ongoing treatment led to a significant improvement in depressive symptoms for those patients with MDD[128]. However, the two subjects with BPD experienced transient hypomanic/manic symptoms. Further studies are necessary to clarify the role of creatine in the mitochondrially mediated pathophysiology of BPD[129].

## FINAL REMARKS

Here we have described potentially promising targets for the development of new, improved treatments for mood disorders. Many recent studies have investigated diverse targets/compounds in both animal models and at the proof-of-concept stage. These include: (1) glycogen synthase kinase-3 (GSK-3) and protein kinase C (PKC), (2) the purinergic system, (3) histone deacetylases (HDACs), (4) the melatonergic system, (5) the tachykinin neuropeptides system, (6) the glutamatergic system, and (7) oxidative stress and bioenergetics. Several promising compounds targeting these systems have either already undergone or are currently undergoing clinical trials in mood disorders. These include glutamatergic modulators, PKC inhibitors, allopurinol, agomelatine, and neurokinin receptor antagonists.

It is important to note that none of these new treatments are FDA approved for the treatment of mood disorders. Also, important differences exist when comparing specific compounds, such as patient cohort and methodological aspects. Despite the many recent advances in our knowledge, future studies investigating the efficacy, safety, and potential mechanisms involved in faster antidepressant and antimanic actions targeting these systems are necessary. A better understanding of the neurobiological underpinnings of BPD, informed by preclinical and clinical research, is essential for the future development of targeted therapies that are more effective, act more rapidly, and are better tolerated than currently available treatments. Such novel and improved therapeutics would have a vast and considerable impact on public health worldwide.

## DISCLOSURES

This research was supported by the Intramural Research Program of the National Institute of Mental Health (NIMH). The authors declare that there are no personal financial holdings that could be perceived as constituting a potential conflict of interest. Dr. Zarate is listed as a coinventor on a patent for the use of ketamine in major depression; he has assigned his patent rights on ketamine to the U.S. Government.
